# HCV Genotypes, Characterization of Mutations Conferring Drug Resistance to Protease Inhibitors, and Risk Factors among Blood Donors in São Paulo, Brazil

**DOI:** 10.1371/journal.pone.0086413

**Published:** 2014-01-21

**Authors:** Anna S. Nishiya, Cesar de Almeida-Neto, Suzete C. Ferreira, Cecília S. Alencar, Claudia Di-Lorenzo-Oliveira, José E. Levi, Nanci A. Salles, Alfredo Mendrone, Ester C. Sabino

**Affiliations:** 1 Fundação Pró-Sangue/Hemocentro de São Paulo, São Paulo, Brazil; 2 Infectious Diseases Division (DIPA), Federal University of São Paulo, São Paulo, Brazil; 3 Discipline of Medical Science, Faculty of Medicine, University of São Paulo, São Paulo, Brazil; 4 LIM 03 Lab. Medice Laboratory, Department of Pathology, HCFMUSP, São Paulo, Brazil; 5 University of São João del-Rei, Divinópolis, Minas Gerais, Brazil; 6 Department of Infectious Disease, Faculty of Medicine, University of São Paulo, São Paulo, Brazil; University of California Merced, United States of America

## Abstract

**Background:**

Hepatitis C virus (HCV) infection is a global health problem estimated to affect almost 200 million people worldwide. The aim of this study is to analyze the subtypes and existence of variants resistant to protease inhibitors and their association with potential HCV risk factors among blood donors in Brazil.

**Methods:**

Repeat anti-HCV reactive blood donors are systematically asked to return for retest, notification, and counseling in which they are interviewed for risk factors for transfusion-transmitted diseases. We analyzed 202 donors who returned for counseling from 2007 to 2010 and presented enzyme immunoassay- and immunoblot-reactive results. The HCV genotypes and resistance mutation analyses were determined by the direct sequencing of the NS5b and NS3 regions, respectively. The HCV viral load was determined using an in-house real-time PCR assay targeting the 5′-NCR.

**Results:**

HCV subtypes 1b, 1a, and 3a were found in 45.5%, 32.0%, and 18.0% of the donors, respectively. The mean viral load of genotype 1 was significantly higher than that of the genotype 3 isolates. Subtype 1a was more frequent among young donors and 3a was more frequent among older donors. Protease inhibitor-resistant variants were detected in 12.8% of the sequenced samples belonging to genotype 1, and a higher frequency was observed among subtype 1a (20%) in comparison to 1b (8%). There was no difference in the prevalence of HCV risk factors among the genotypes or drug-resistant variants.

**Conclusions:**

We found a predominance of subtype 1b, with an increase in the frequency of subtype 1a, in young subjects. Mutations conferring resistance to NS3 inhibitors were frequent in treatment-naïve blood donors, particularly those infected with subtype 1a. These variants were detected in the major viral population of HCV quasispecies, have replicative capacities comparable to nonresistant strains, and could be important for predicting the response to antiviral triple therapy.

## Introduction

HCV was identified by Choo et al. in 1989 [1] and is currently a major cause of chronic hepatitis in the world, with almost 200 million carriers (2–3% of the global population) and approximately 350,000 deaths annually [2].

Blood transfusion, contaminated blood products, and unsafe medical practices were the main routes of global HCV spread after the Second World War until the early 1980s [3]. Although the introduction of screening tests for blood donors reduced the risk of transfusion-transmitted HCV [4], the use of illicit intravenous drugs remains one of the major risk factors for HCV infection [5]. Sexual behavior can also be a minor risk factor for infection yet can be an important route of transmission among intravenous drug users (IVDUs), particularly men who have sex with other men or prostitutes [6], [7]. In Brazil, the prevalence of HCV infection may reach 11% in IVDUs [8], [9], is approximately 1.5% in the general population [10]–[12], and ranges from 0.21% to 1.1% in blood donors [13]–[18]. Furthermore, HCV prevalence is decreasing among blood donors, and de Almeida-Neto et al. found a low HCV prevalence (0.19%) in three large Brazilian blood centers in 2013, which could be due to improvements in blood donor selection and in the social and economic conditions of the population [19].

HCV has a high genetic variability and is classified into seven major genotypes and over 100 subtypes that vary with regard to their geographic distribution, risk factors associated with infection, and response to treatment [20]–[22]. The combination of pegylated interferon alpha (IFN-α) (an important mediator of the innate antiviral immune response) and ribavirin (a nucleoside analog that acts on viral replication) was the standard treatment of patients with chronic hepatitis C during many years [23], [24]. Genotypes 1, 2, and 3 are widely distributed [21], [25], [26]; genotype 1 is the most prevalent and also presents the worst response to antiviral therapy, with only 40% of patients responding to this treatment [27], [28]. Thus, in the search for more effective drugs, new direct-acting inhibitors have recently been developed to inhibit the nonstructural protein (NS3/4) of genotype 1, a protease that is important for the cleavage of the polyprotein during the viral replicative cycle [29]. Two first-generation drugs, boceprevir and telaprevir, were approved for use in the USA and Europe in 2011 [30]–[32] and in Brazil is provided at no cost for the treatment of genotype 1-infected patients with advanced fibrosis or cirrhosis [33]. In preliminary studies, these protease inhibitors alone induced the rapid selection of resistant viral variants [34], [35]; however, the combination of these new drugs that inhibit the viral protease (boceprevir or telaprevir) with INF-α and ribavirin (triple therapy) showed an improvement in the sustained virological response (SVR) rate of up to 75% [30], [31], [36]–[38].

Despite the success of direct-acting drugs, approximately 25% of patients infected with genotype 1 do not achieve SVR [30], [31]. During treatment, viral breakthrough can occur in approximately 2–7% of patients in whom there is the reappearance of HCV RNA, mainly due to the emergence of higher-level resistant variants, which explains the failure of therapy [30], [39], [40].

The variants associated with resistance are present prior to therapy and are part of the quasispecies population in infected individuals. These mutations, which do not alter viral fitness, may occasionally be found in frequencies ranging from 2–29% in untreated individuals [41]–[49]. The major reason for this wide variation in frequency depends on which genotype/subtypes are studied and how the variants, as potential resistance mutations, are defined. Indeed, baseline resistance variants can vary worldwide from 14–29% in Europe to <1–9% in the USA among patients infected with subtype 1a, with V36L, T54S, V55A, Q80K, and R155K as the main substitutions found. The frequency for subtype 1b in Japan was found to be 5%, in Europe from 10–25%, and approximately 2% in the USA, with V36L and T54S as the frequent mutations [43]–[46], [48]–[50].

In this study, we analyzed HCV subtypes and the existence of variants resistant to protease inhibitors and their association with demographic and potential risk factors involved in the spread of HCV in a group of Brazilian blood donors.

## Materials and Methods

### Ethics Statement

This project was approved by the Ethics Committee of the Federal University of São Paulo (UNIFESP)/São Paulo Hospital under protocol 1760/11. As a routine process of the blood bank, donors sign an informed consent to allow the use of their samples for quality control purposes. The study linked the routine epidemiological data obtained during medical counseling with the genotype and viral load data performed to characterize the sample for the quality control department. The dataset and an aliquot of the sample were anonymized for the data analysis and drug resistance testing.

### Study Population

**Figure 1:**
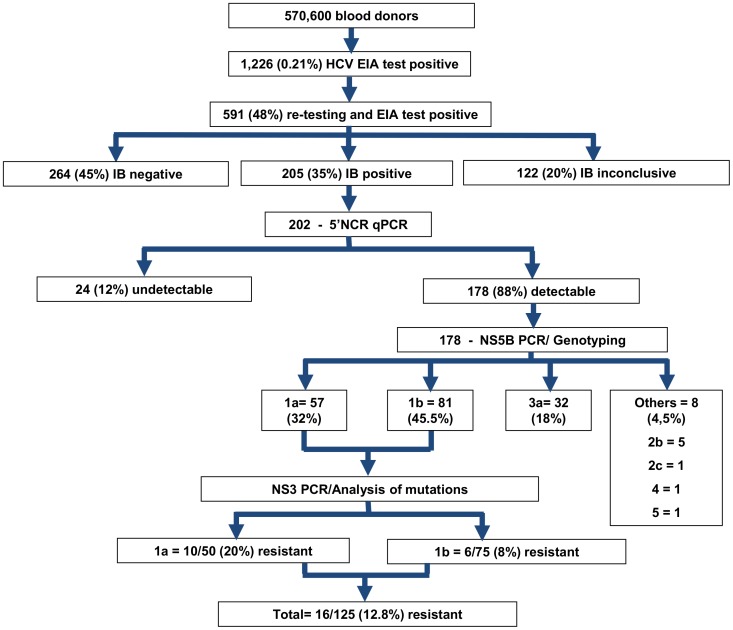
Flowchart summarizing the routine screening and confirmatory testing for HCV and the results of the genotyping and protease inhibitor-resistance mutation analyses. Notes: EIA - Enzyme immunoassay (EIA); IB - Immunoblot assay.

From September 2007 to July 2011, a total of 570,600 blood donors of Fundação Pró- Sangue/Hemocentro de São Paulo (Brazil) were screened for HCV using a high-sensitivity enzyme immunoassay (EIA) to detect antibodies, and 1,226 (0.21%) samples were found to be reactive. Only 591 of these donors (48%) returned for re-testing. The samples from donors with a reactive result for the second EIA were subjected to an immunoblot assay (IB). Of the 591 samples, 205 (35%) were confirmed positive for HCV antibodies by immunoblotting, 264 (45%) were negative, and 122 (20%) were inconclusive. Three samples had no sufficient amount of serum and were withdrawn. Therefore, a total of 202 samples were included in this study (Figure 1). HCV antibodies were screened using ORTHO HCV 3.0 (Biolab-Mérieux S/A, Rio de Janeiro, Brazil), and immunoblot CHIRON RIBA HCV 3.0 S/A (Chiron Corporation, Emeryville, EUA) was used for the diagnostic confirmation.

As a part of the routine counseling process, the donors were systematically asked about potential HCV risk factors involved in transmission by blood transfusion, previous medical procedures, such as surgeries and endoscopies, exposure to contamination through tattooing, piercing, manicure, pedicure, or barber procedures, intravenous drug use, occupational exposure, and sexual behavior. These data were recorded in the information system of the blood bank. Core demographic data, such as age, sex, ethnicity, and education, were also recorded. The data were extracted from the operation system and linked to the sample, and the dataset was anonymized.

### HCV Extraction, cDNA Synthesis, and Determination of Viral Load

The quantitative assay to detect the HCV viral load was performed by an in-house real-time PCR assay that amplified part of the 5′-NCR region (5′ noncoding region) with a sensitivity of approximately 400 IU/mL. Total RNA was extracted from 140 µL of plasma using the QIAamp Viral RNA Mini Kit (Qiagen, Hilden, Germany) and eluted in 60 µL of elution buffer. cDNA was obtained by a reverse transcriptase reaction using 10 µL of the extracted RNA, 15 ng/µL of random primer (Amersham Biosciences, Piscataway, NJ, USA), and 10 U/µL of Super Script TM III reverse transcriptase (Invitrogen, Carlsbad, CA, USA) in a buffer containing 0.25 U/µL of ribonuclease inhibitor (Invitrogen, Carlsbad, CA, USA) and 0.5 mM deoxyribonucleotide triphosphates (Invitrogen, Carlsbad, CA, USA) (final volume of 20 µL). The reaction was incubated at 45°C for 90 minutes. The cDNA (5 µL) was added to 20 µL of TaqMan Master Mix (Applied Biosystems, Foster, CA, USA) and amplified by real-time PCR using the following primers and probe: HCV_sense, 5′CGTCTAGCCATGGCGTAAGTA3′; HCV_anti-sense, 5′GGTTCCGCAGACCACTATGG3′; HCV_Probe, 5′FAM-CCCCCCTCCCGGGAGA-NFQ-3′. The reaction was performed using the StepOne system (Applied Biosystems, Foster, CA, USA) for 45–50 cycles with the following program: 10 minutes at 95°C, followed by 45 cycles of 15 seconds for 94°C and 60 seconds at 60°C.

### HCV Phylogenetic Analysis and Genotyping

Only samples with a detectable viral load were subjected to nested PCR to amplify a 400-bp fragment from the non-structural region 5B (NS5B) gene, including part of the RNA-dependent RNA polymerase, using previously described PCR primers and conditions [51], [52]. The PCR product was purified using the QIAquick kit (QIAGEN, Hilden, Germany) and directly sequenced using the Big Dye Terminator Cycle Sequencing Ready Reaction (Applied Biosystems, Foster City, USA) according to the manufacturer’s procedure.

The sequences were edited using the SEQUENCHER program (Gene Codes Corporation Ann Arbor, MI, USA) and aligned with the reference sequences of the six main genotypes obtained from GenBank in Bioedit [53]. The phylogenetic analysis was performed using the PHYLIP 3.5c program [54]. Bootstrap values were determined on 1000 replicates of the sequence data with the SEQBOOT program (PHYLIP 3.5c). The phylogenetic relationships were constructed using the neighbor-joining method, and the pairwise distances were calculated by DNADIST (PHYLIP 3.5c). A consensus tree was identified using the CONSENSE program (PHYLIP 3.5c).

Genotype confirmation was performed using available online tools at the following sites: (http://www.bioafrica.net/regagenotype/html/subtypinghcv.html) and (http://www.ncbi.nlm.nih.gov/projects/genotyping/formpage.cgi).

The HCV sequences were deposited in GenBank under accession numbers KF523955–KF524257.

### Analysis of Mutations Associated with Resistance to Protease Inhibitors

All the genotype 1 samples were subjected to nested or semi-nested PCR to amplify the region encoding the non-structural region 3 (NS3) protease catalytic domain [34], [55], [56] and sequenced as described above. Predictions of the phenotypic resistance of the NS3 region to boceprevir and telaprevir were determined using the online tool geno2pheno [hcv] (http://hcv.bioinf.mpi-inf.mpg.de/index.php) [56], [57]. This algorithm considers amino acid substitutions associated with resistance observed in clinical trials (V36A/G/L/M, Q41R/K/P, F43C/S/L, T54A/S, V55A, Q80R/K, R109K, R155K/T/G/I/M/S, A156S/T/V, and V170A/T/G) [49], [58]–[61] and others rarely reported in vivo (A87T, R117H, N174F, and S138T/D) [62]. For a ‘next-generation’ drug (simeprevir), the sequences were aligned with a reference in Bioedit [53], and the amino acid mutations were manually verified (V36M, F43S, T54S, Q80K/R/L, S122A/R, S138T, R155K, A156TV, and D168A/V/E/H/T) [62].

### Statistical Analysis

The characteristics were evaluated according to the genotype or resistance profile of the patients. A chi-square test was used to compare the proportions at a 95% significance level. Tukey’s t test was used to compare the means.

## Results

Among 202 HCV-positive samples from blood donors, 88% (178/202) were positive for HCV RNA and showed the following viral load distribution: 3.4% (6/178) with viral loads between 400 and 9,999 IU/mL, 11.8% (21/178) between 10,000 and 99,999 IU/mL, 29.8% (53/178) between 100,000 and 999,999 IU/mL, and 55.0% (98/178) with>1,000,000 IU/mL. A total of 12% (24/202) of donors had an undetectable viral load.

The HCV genotypes were determined by NS5b amplification, and sequencing was successful in all 178 RNA-detectable samples; of these, 45.5% (81/178) were typed as 1b, 32.0% (57/178) as 1a, 18.0% (32/178) as 3a, and 4.5% (8/178) as others (2b (5/8), 2c (1/8), 4 (1/8), and 5 (1/8)) (figure 1).

The genotypes were not associated with gender, ethnicity, or educational level; however, subtype 1a was more frequently found among young donors, 1b among the intermediate age group, and subtype 3a among older donors (p = 0.047) (Table 1). Viral load was not associated with gender or age, but genotype 3a (5.22 log_10_ IU/mL) had a lower log mean than genotype 1a (5.99 log_10_ IU/mL) (3a vs 1a, p = 0.0002) and genotype 1b (6.35 log_10_ IU/mL) (3a vs 1a; p = 0.0000) (Table 1). There was no difference in the prevalence of all risk factors among the different genotypes, despite the high frequency of donors that cited the following: previous blood transfusion, 11.2% (20/178); intravenous drug use, 6.4% (10/155); and have given or received money or drugs for sex, 12.8% (20/156).

**Table 1:** HCV genotype distribution according donor demographics, risk factors, and HCV viral load.

		Subtype (%)				
Characteristics	Subcategory	1a n = 57	1b n = 81	3a n = 32	Others n = 8	p-value
**Sex**	Male	29 (28.7)	51 (50.5)	18 (17.8)	3 (3.0)	0.45*
	Female	28 (36.4)	30 (39.0)	14 (18.2)	5 (6.4)	
**Age**	18–29 y	19 (42.2)	17 (37.8)	7 (15.5)	2 (4.5)	0.047*
	30–39 y	15 (26.8)	34 (60.7)	6 (10.7)	1 (1.8)	
	40–49 y	16 (35.6)	17 (37.8)	11 (24.4)	1 (2.2)	
	>50 y	7 (21.9)	13 (40.6)	8 (25.0)	4 (12.5)	
**Ethnicity**	Mulatto	7 (26.9)	16 (61.5)	2 (7.7)	1 (3.9)	0.39*
	Pardo	22 (32.8)	30 (44.8)	13 (19.4)	2 (3.0)	
	White	27 (35.2)	31 (40.3)	14 (18.2)	5 (6.5)	
	Asian	0 (0.0)	1 (50.0)	1 (50.0)	0 (0.0)	
	Missing	1	3	2	0	
**Education**	<8 y	7 (20.6)	16 (47.1)	8 (23.5)	3 (8.8)	0.77*
	Completed 8 y	8 (42.1)	8 (42.1)	3 (15.8)	0 (0.0)	
	11 y	33 (37.9)	38 (43.7)	13 (14.9)	3 (3.5)	
	Technical college	2 (40.0)	2 (40.0)	1 (20.0)	0 (0.0)	
	University course	6 (24.0)	13 (52.0)	4 (16.0)	2 (8.0)	
	Missing	1	4	3	0	
**Blood transfusion**	Yes	5 (25.0)	8 (40.0)	7 (35.0)	0 (0.0)	0.26*
	No	52(32.9)	73(46.2)	25 (15.8)	8 (5.1)	
**Tattoo**	Yes	11 (34.4)	14 (43.7)	4 (12.5)	3 (9.4)	0.81*
	No	36 (30.0)	54 (45.0)	25 (20.8)	5 (4.2)	
	Missing	10	13	3	0	
**Occupational exposure**	Yes	4 (44.4)	4 (44.4)	1 (11.2)	0(0.0)	0.57*
	No	46 (31.3)	65 (44.2)	28 (19.1)	8 (5.4)	
	Missing	7	12	3	0	
**Sex partner of IVDU**	Yes	0 (0.0)	5 (83.3)	1 (16.7)	0 (0.0)	0.050*
	No	45 (31.0)	64 (44.2)	28 (19.3)	8 (5.5)	
	Missing	12	12	3	0	
**Sex partner of hepatitis+ individual**	Yes	1 (33.4)	1 (33.3)	0 (0.0)	1 (33.3)	0.49*
	No	48 (31.6)	68 (44.7)	29 (19.1)	7 (4.6)	
	Missing	8	12	3	0	
**Have given or received** **money or drugs for sex**	Yes	4 (20.0)	10 (50.0)	5 (25.0)	1 (5.0)	0.77*
	No	46 (33.8)	59 (43.4)	24 (17.6)	7 (5.2)	
	Missing	7	12	3	0	
**UDIV**	Yes	2 (20.0)	7 (70.0)	0 (0.0)	1 (10.0)	0.37*
	No	48 (33.1)	62 (42.8)	28 (19.3)	7 (4.8)	
	Missing	7	12	4	0	
**HBV+ or HCV+ relatives**	Yes	5 (19.3)	15 (57.7)	3 (11.5)	3 (11.5)	0.10*
	No	45 (34.6)	54 (41.5)	26 (20.0)	5 (3.9)	
	Missing	7	12	3	0	
**HCV RNA (log_10_ UI/mL) mean**	Mean	5.99	6.35	5.22	6.64	
	Subtype 3a vs 1a					0.0002**
	Subtype 3a vs 1b					0.0000**

* Pearson’s chi-squared test.

** Tukey’s t test.

We found that 19.1% (34/178) of the donors reported no known risk factors associated with infection; 19.1% (34/178) reported only one potential parenteral or sexual exposure, 32.0% (57/178) reported two factors, and 29.8% (53/178) reported three or more (multiple) risk factors.

Only the genotype 1 samples were analyzed by amplification and sequencing of the region encoding the NS3 viral protease, which was successful in 90.6% (125/138) or 88.0% (50/57) cases of subtype 1a and 93.0% (75/81) cases of subtype 1b. The results of genotyping by phylogenetic analysis or online analysis tools assigned the same genotype.

The frequency of blood donors with a resistance-associated variant was 10.4% (13/125) for boceprevir, 11.2% (14/125) for telaprevir, and 6.4% (8/125) for simeprevir, for a total of 16/125 (12.8%) for all three protease inhibitors (Table 2).

**Table 2:** Amino acid substitutions and frequency of blood donors with mutations conferring resistance to protease inhibitors.

Sample n^o^	Subtype	Mutation	Resistance
6	1a	V55A	Boceprevir/Telaprevir
32	1a	R155K	Boceprevir/Telaprevir/Simeprevir
33	1b	R117H	Boceprevir/Telaprevir
44	1a	R155K	Boceprevir/Telaprevir/Simeprevir
54	1b	D168G	Boceprevir
55	1a	V36L/V55A	Boceprevir/Telaprevir
62	1a	R155K	Boceprevir/Telaprevir/Simeprevir
63	1b	T54S	Telaprevir/Simeprevir
64	1a	Q80L	Low Simeprevir
70	1b	R117H	Boceprevir/Telaprevir
93	1a	R155K	Boceprevir/Telaprevir/Simeprevir
107	1a	V55A	Boceprevir/Telaprevir
110	1a	V36L	Boceprevir/Telaprevir
127	1b	V55A	Boceprevir/Telaprevir
134	1b	T54S	Telaprevir/Simeprevir
184	1a	R155K	Boceprevir/Telaprevir/Simeprevir
Totalfrequency			16/125 (12.8%)
Boceprevir			13/125 (10.4%)
Telaprevir			14/125 (11.2%)
Simeprevir			8/125 (6.4%)

Drug-associated mutations were found at a higher frequency in individuals infected with subtype 1a (20%) than subtype 1b (8%) (p = 0.02) (Table 3). The frequency of mutations associated with protease inhibitor resistance was 20% (10/50) among the subtype 1a isolates, with the following changes: [(2x) V36L, (3x) V55A, (1x) Q80L, and (5x) R155K]. For subtype 1b, the frequency of these mutations was 8% (6/75), with the following substitutions: [(2x) T54S, (1x) V55A, (2x) R117H, and (1x) D168G]. The most prevalent mutation, R155K, was found in 4.0% (5/125) of all the sequences or 10% (5/50) of the subtype 1a sequences.

**Table 3:** Frequency of blood donors with mutations conferring resistance to protease inhibitors according to demographics, risk factors, subtypes, and HCV viral load.

		Total n = 125	N (%) of resistance n = 16	p-value
**Sex**	Male	75	9 (12.0)	0.37*
	Female	50	7 (14.0)	
**Age**	18–29 y	33	5 (15.2)	0.30*
	30–39 y	45	4 (8.9)	
	40–49 y	28	6 (21.4)	
	>50 y	19	1 (5.3)	
**Ethnicity**	Pardo	68	8 (11.8)	0.90*
	White	53	7 (13.2)	
	Asian	1	0 (0.0)	
	Missing	3	1	
**Education**	<8 years	20	1 (5.0)	0.58*
	Completed 8 Years	16	3 (18.8)	
	11 years	66	8 (12.1)	
	Technical college	3	1 (33.3)	
	University course	16	2 (12.5)	
	Missing	4	1	
**Blood transfusion**	Yes	13	1 (7.7)	0.28*
	No	112	15 (13.4)	
**Tattoo**	Yes	25	1 (4.0)	0.10*
	No	78	10 (12.8)	
	Missing	22	5	
**Occupational exposure**	Yes	5	1 (20.0)	0.29*
	No	101	12 (11.9)	
	Missing	19	3	
**Sex partner of blood transfusion recipient**	Yes	2	1 (50.0)	0.05*
	No	103	12 (11.6)	
	Missing	20	3	
**Condom use**	Yes	36	4 (11.1)	0.33*
	No	64	9 (14.1)	
	Missing	25	3	
**Have given or received** **money or drugs for sex**	Yes	13	2 (15.4)	0.35*
	No	93	11 (11.8)	
	Missing	19	3	
**IVDU**	Yes	9	1 (11.1)	0.45*
	No	97	12 (12.2)	
	Missing	19	3	
**HBV+ or HCV+ relatives**	Yes	19	4 (21.1)	0.09*
	No	87	9 (10.3)	
	Missing	19	3	
**HCV Subtype**	1a	50	10 (20.0)	0.02*
	1b	75	6 (8.0)	
**HCV RNA (log 10 UI/mL) mean**	Resistant variant	6.06		0.8485**
	Wild type	6.32		

* Pearson;s chi-squared test.

** Tukey’s t test.

There was no association of drug resistance with sex, ethnicity, education level, age, or any risk factor. In addition, no difference in the log mean viral load between the resistant variants and wild type was observed (6.06 log_10_ IU/mL vs 6.32 log_10_ IU/mL, p = 0.8485) (Table 2). Moreover, drug resistance could not be associated with any risk factor (Table 3).

## Discussion

HCV subtype 1b was the most prevalent subtype in our studied blood donors and was found in almost half of the cases, followed by subtypes 1a and 3a. We also found such rare genotypes as 2b, 2c, 4, and 5 at very low frequencies. These data are in agreement with Brazilian studies that have shown a predominance of subtype 1a or 1b, followed by 3 in patients from the state of São Paulo [63]–[65].

Most donors exhibited a very high viral load. When analyzing the viral load according to the subtype distribution, subtype 3a presented a lower mean viral load than subtypes 1a and 1b. Previous studies have also described an association between genotype 1 and higher levels of HCV-RNA in the serum of infected patients compared to those infected with genotypes 2 and 3 [66]–[68]. Viral load was undetectable in 12% of the donors, and half of them were female. It is known that spontaneous viral clearance may occur in approximately 25% of HCV infections, mainly in women and some ethnic groups in association with the favorable genotype of the IL28B gene [69]–[71].

The genotype distribution according to age showed that subtype 1a was more frequent among younger donors and subtype 3a in donors older than 40 years. This result differs in part from another study also performed in São Paulo that reported that subtypes 1a and 3a appear to be more associated with newly infected young people and possibly with a higher rate of sexual transmission [65]. Both genotypes were also associated with IVDUs [72], [73] and presented a high prevalence in this group, reaching 60% for subtype 1a and 20% for 3 [8], [74]. Moreover, Vigani et al. [75] reported an association between genotype 3 and the non-illicit IV drug Gluconergan, which was commonly used in the 1970s in São Paulo as a stimulant. Indeed, Gluconergan users shared needles and syringes, and the IV route might explain the high frequency of HCV genotype 3 among older blood donors.

Subtype 1b began to spread 40–60 years earlier than subtype 1a. The spread of subtype 1b has been associated with blood transfusions during the 1940s until the 1980s and affected a wide age range [76], which explains the high frequency of this subtype in the intermediate age group of our donors. Despite being the most prevalent in our population, subtype 1b has shown a slower growth rate than genotypes 1a and 3a [65], and perhaps a change in the prevalence in the predominant genotype may occur in Brazil, as has been verified in the USA and Europe [77], [78]. The probable hypothesis is that the introduction of new subtypes in high-risk groups may rapidly impact lower-risk groups by an increasing prevalence in the general population [78].

There was no association of any genotype with potential HCV risk factors, such as IVDU, blood transfusion, or sexual behavior. This finding suggests that risk factors for HCV transmission are still unknown in our population or that different genotypes are disseminating through different transmission routes in blood donors. Approximately 80% of the infected patients mentioned at least one HCV risk factor, whereas 20% of the donors did not disclose any risk factor. A large proportion of donors had multiple risk factors associated with HCV transmission, and it is possible that the sum of several factors may be contributing to the spread of the virus. In our study, a single individual reported tattooing, piercing, surgery, endoscopy, transfusion, manicure, and highly risky sexual behavior, which depicts a common scenario in our current society.

After mandatory screening for anti-HCV antibodies, the risk of transmission by blood transfusions has decreased markedly and is currently very low (5/1,000,000 units transfused) [19], similar to the incidence in some locations in Europe and the USA. Nonetheless, measures to prevent HCV transmission among drug users are needed and also in medical and paramedical practices. In addition, special attention to high-risk sexual behavior among UDIV infected or co-infected with HIV is warranted.

Brazilian studies have found frequencies of between 3.2% and 18.9% of patients with variants associated with resistance among HCV chronic carriers not treated with protease inhibitors [41], [42], [79]. Our data showed an intermediate total frequency of resistance when compared to other results found in Brazil.

Among the genotype 1a-infected Brazilian donors, we observed the mutations V36L (4%) and V55A (6%), which confer a low level of resistance at frequencies close to those found the USA (1–2% for V36M) and Europe (3–6% for V36L and V55A) [44]–[46], [48]–[50]. In contrast, a prevalence different from that found in other studies was identified for two mutations, Q80K and R155K, which confer moderate-level resistance. The Q80K mutation, which is associated with resistance to simeprevir and is very common in the USA (40%) [50] and Europe (4–16%) [45], [46], was not found in our study. Another important observation was that the R155K mutation has a low prevalence in the USA (1%) [48]–[50] but was found in 10% of the untreated individuals in our population. These findings could be related to certain Brazilian 1a subtypes, and it appears that the Brazilian sequences have their own pattern of mutations and frequencies, which differ from other parts of the world.

Next-generation drugs have been developed to be effective in different genotypes and have a higher genetic barrier to resistance than first-generation drugs [62] Indeed, we observed a lower frequency of baseline variants resistant to simeprevir (6%) than boceprevir (10%) and telaprevir (11%), and these data could help in future treatment choices for the Brazilian population.

The genetic barrier for resistance to protease inhibitors may also vary according to different HCV subtypes. The frequency of resistance mutations was twice as high among patients with subtype 1a compared to those with subtype 1b. An amino acid substitution at position R155K requires only a single nucleotide change (a transition) to confer resistance, whereas mutation for subtype 1b requires two nucleotide changes, one of which must be a transversion [80]. Pawlotsky et al. [59] reported a higher frequency of virologic failure for subtype 1a due to the lower genetic barrier to viral resistance than for subtype 1b. The R155K mutation also confers cross-resistance to all protease inhibitors though does not modify the fitness of the strains that carry this mutation. Our study showed no difference between the viral loads of resistance or sensitivity of the strains, confirming that both have comparable replicative capacities. We should emphasize that, as the classical sequencing techniques can identify the major viral population, the variants detected could be important with regard to treatment failure.

In summary, we report a predominance of subtype 1b, with an increase in the frequency of subtype 1a in younger donors. Rare genotypes, such as 2b, 2c, 4, and 5, were also found. HCV subtypes 1a and 1b showed higher viral loads than subtype 3a. We failed to associate any genotype to potential HCV risk factors; known risk factors explained 80% of the infected cases, but 20% of the donors did not report any of the risk factors examined. Regardless, the majority of donors reported more than one risk factor for HCV infection. Thus, blood banks must be encouraged to increase notification and counseling measures for these donors to prevent the spread of HCV. Mutations conferring resistance to NS3 inhibitors were frequent in treatment-naïve blood donors but could not be associated with any risk factor. The presence of these mutations in the major population of HCV quasispecies that have replicative capacities comparable to nonresistant strains could be important for predicting response to antiviral triple therapy.
